# Adipose inflammation: South Asian ethnicity and central obesity are independently associated with higher immune cell recruitment to adipose‐specific media: A pilot study in men

**DOI:** 10.14814/phy2.15883

**Published:** 2023-11-27

**Authors:** Matthew J. Roberts, Malik Hamrouni, Alex J. Wadley, Nicolette C. Bishop

**Affiliations:** ^1^ National Centre for Sport and Exercise Medicine, School of Sport, Exercise and Health Sciences Loughborough University Loughborough UK; ^2^ National Institute for Health Research (NIHR) Leicester Biomedical Research Centre University Hospitals of Leicester, National Health Service (NHS) Trust and the University of Leicester Leicester UK; ^3^ School of Sport, Exercise and Rehabilitation Sciences, College of Life and Environmental Sciences University of Birmingham Birmingham UK

**Keywords:** ethnicity, inflammation, monocytes, obesity

## Abstract

A South Asian (SA) cardiovascular phenomenon exists whereby SAs have excess burden of cardiovascular disease (CVD) despite having low prevalence of recognized CVD risk factors. The aim of the current study was to determine whether perturbations in monocyte biology contribute to this phenomenon via higher circulating cell numbers, a more pro‐inflammatory phenotype, and higher transmigration and adhesion. Adhesion is linked to vascular inflammation whereas transmigration is linked to tissue inflammation. SA men with (*N* = 10; SAs with central obesity [CO‐SA]) and without (*N* = 10; lean SA [LE‐SA]) central obesity, plus White European counterparts (*N* = 10; white Europeans with central obesity [CO‐WE], *N* = 10; lean white Europeans [LE‐WE]) participated. An ex vivo assay mimicking blood flow dynamics coupled to flow cytometry determined the adhesion and transmigration of monocyte subsets toward chemokine‐rich media cultured from pre‐adipocytes (absolute responses). Migration and adhesion were also standardized for differences in numbers of circulating monocytes between participants (relative responses). Metabolic and inflammatory markers were assessed. SAs had higher absolute (but not relative) adhesion and migration of monocytes than WEs. Central obesity was associated with higher absolute and relative adhesion and migration of monocytes. SAs had higher concentrations of all monocyte subsets compared with WEs coinciding with adverse cardiovascular‐inflammatory profiles. LE‐SAs had similar monocyte concentrations, transmigration, and adhesion compared with CO‐WEs, corresponding with similar cardiovascular‐inflammatory profiles. The study provides novel evidence for higher monocyte counts associated with higher transmigration and adhesion in SA compared with WE men. Importantly, similar monocyte biology and cardiovascular‐inflammatory profiles were seen in LE‐SAs compared with CO‐WEs, which may contribute to the higher risk of CVD at lower body mass index experienced by SAs.

## Graphical abstract

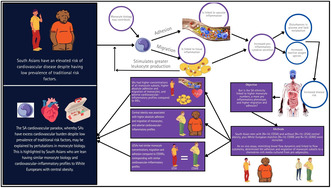

A graphical abstract highlighting the rationale, methods, results, and conclusion of the study.

## 1 INTRODUCTION

Atherosclerotic cardiovascular disease (CVD) is the primary cause of death globally (Tsao et al., 2022) and South Asians (SAs—people of Indian subcontinent descent) are disproportionately affected (Volgman et al., 2018). Compared with white Europeans (WEs), ethnic SAs living in Europe have a 40% higher risk of coronary heart disease, a two‐ to fourfold higher risk of developing type 2 diabetes mellitus, and experience myocardial infarctions 5–10 years earlier and at a lower body mass index (BMI) (Sattar & Gill, 2015). The heightened CVD risk in SA men compared with WE men is greater than that for SA women to compared with WE women (Chambers et al., 2001), making SA men a group of particular concern.

The term “SA CVD phenomenon” has been used in the literature, to describe the apparent anomaly whereby SAs have a higher risk of CVD even though they have a lower prevalence of recognized risk factors such as elevated BMI, dietary fat intake, hypercholesterolemia, blood pressure, and low‐density lipoprotein cholesterol (Ahmed & El‐Menyar, 2015; Gupta & Brister, 2006). Consequently, the SA ethnicity, in South Asia or after migration, has been suggested to be an independent risk factor for CVD (Gupta & Brister, 2006). More recent research suggested higher relative concentrations of ectopic fat in SAs was a key contributor, but data, consistent across two independent cohorts, presented a limited number of ethnic differences in the distribution of body fat depots associated with metabolic disease (Alenaini et al., 2020). Therefore, other mechanisms must contribute to the heightened CVD risk in SAs.

One such mechanism may be monocyte biology including adhesion and transmigration, but this has not been studied in SAs. In rodent studies and White populations, higher numbers of circulating monocytes, adhesion to the vascular wall, and transmigration to adipose tissue contribute to heightened CVD risk through chronic low‐grade inflammation underpinned by M1 (inflammatory) macrophage accumulation in adipose tissue, intracellular stress, and pro‐inflammatory cytokine release (Bishop et al., 2022). Persistent monocyte tissue migration from the peripheral circulation further stimulates bone marrow hematopoiesis, resulting in chronically high monocyte counts and a sustained pro‐inflammatory cycle (Idzkowska et al., 2015). The local effects spill over systemically contributing to wider organ dysfunction, vascular inflammation, and CVD (Bishop et al., 2022).

Monocyte subsets also exist and can be classified according to their expression of cell surface markers CD14 and CD16. Classical (CM; CD14++CD16‐) are most common (80–90%), with intermediate (IM; CD14++CD16+) and non‐classical (NCM; CD14+CD16++) monocytes contributing 2–5% and 5–10%, respectively (Ziegler‐Heitbrock et al., 2010). IM and NCM are a major source of the inflammatory cytokines TNF‐alpha (TNF‐α) and interleukin‐6 (IL‐6), and proportions of IM and NCM are elevated with obesity and other inflammatory diseases including CVD (Bishop et al., 2022).

We have shown that people with central obesity—but otherwise healthy—had greater concentrations of CM, IM, and NCM in the blood, and in turn, higher ex vivo migration (mimicking transmigration into tissue) and adhesion (mimicking adhesion to the vascular wall) than people who were lean (Wadley et al., 2021). Others have recently reported that SA patients at risk of coronary atherosclerosis have higher proportions of IM and NCM compared with WEs (Ramanathan et al., 2019). However, no studies have investigated how higher numbers of monocytes in the peripheral circulation may be directly implicated in higher CVD risk for SAs. It is therefore reasonable, yet unknown, that SAs have higher risk of CVD underpinned by a cascade of perturbations in monocyte biology, higher numbers in peripheral blood, a more pro‐inflammatory phenotype, and greater transmigration and adhesion from the periphery into adipose tissue.

Therefore, the present study used a dynamic model replicating human physiological blood flow conditions, coupled with flow cytometric analysis to investigate (1) whether SAs with and without central obesity have higher adhesion and migration of monocytes compared to their respective WE counterparts (2) if central obesity is associated with higher adhesion and migration of monocytes in SAs (3) if the magnitude of the response is greater in SAs with central obesity versus lean SAs, compared to WEs with central obesity versus lean WEs.

## 2 METHODS

### 2.1 Ethical approval and participant recruitment

Ethical approval was granted by the institutional Ethics sub‐committee and registered at ClinicalTrials.gov (NCT04761081). The study was pre‐registered with a recruitment target of 40 participants as a pragmatic sample size due to substantial analysis costs and this being a pilot study to generate much needed data to justify larger studies of monocyte biology and CVD risk in specific SA populations. Written informed consent was obtained from 40 men (10 lean white Europeans [LE‐WE], 10 white Europeans with central obesity [CO‐WE], 10 lean South Asians [LE‐SA], 10 SAs with central obesity [CO‐SA]). To establish ethnicity, participants completed a verification form as reported previously (Arjunan et al., 2015). All WEs were North‐western Europeans (White British or White French) and currently living in the United Kingdom. All SA participants were currently living in the United Kingdom but had parents or grandparents born in South Asia. Central obesity was defined as a waist circumference ≥94 cm in WEs and ≥90 cm in SAs according to the International Diabetes Federation cut points (Alberti et al., 2007). Participants were matched between groups for age, were non‐smokers, not taking any anti‐inflammatory medications and were free from CVD.

### 2.2 Experimental procedures

Participants refrained from strenuous physical activity (confirmed via an ActiGraph GT3X activity monitor) and avoided caffeine and alcohol for 48 h prior to the experimental session. Participants arrived at the laboratory at 08:00, having fasted from 22:00 (except plain water) the previous evening. Height and weight were measured using a fixed wall stadiometer with a digital weighing scale built in (Seca Ltd, Hamburg, Germany). Hip and waist circumference were measured using a flexible, non‐elastic tape (Hokanson, Washington, USA) using established measurement guidelines (Alberti et al., 2007). Body fat percentage was determined using bioelectrical impedance analysis (Seca mBCA 515, Seca Ltd, Hamburg, Germany). A blood sample was then collected via venepuncture to an antecubital vein into EDTA (1 × 4.9 mL) and sodium heparin‐coated (4 × 7.5 mL) monovettes.

### 2.3 Blood analysis

A full blood count was performed on EDTA‐treated whole blood using a hematology analyzer (Yumizen H500, Horiba, Northampton, UK). The remaining EDTA‐treated blood was centrifuged at 3500*g* for 10 min at 4° to isolate plasma that was stored at −80°C for future analysis. Triacylglycerol (TAG), total cholesterol (TC), high‐density lipoprotein (HDL), low‐density lipoprotein (LDL), glucose, and C‐reactive protein (CRP) concentrations were determined spectrophotometrically using commercially available kits and a benchtop analyzer (Pentra 400, Horiba Medical, Montpellier, France, catalog numbers: TAG:1220001640, TC:1220001634, HDL:1220001636, LDL:1220001638, glucose:1220001668, CRP:1220001611). Plasma insulin (Mercodia, Uppsala, Sweden, catalog number: 10‐1113‐01) and TNF‐α (high sensitivity) (R&D Systems, Abingdon, UK, catalog number: HSTA00E) were determined using commercially available enzyme‐linked immunosorbent assays. Homeostatic model assessment for insulin resistance (HOMA‐IR) was calculated using the formula: HOMA‐IR = (glucose [mmol/L] × insulin [mU/L])/22.5.

### 2.4 Ex vivo migration assay

Peripheral blood mononuclear cells (PBMCs) were separated from heparinized blood by density gradient centrifugation as reported previously (Wadley et al., 2021). PBMC counts were quantified using CountBright™ Absolute Counting Beads (ThermoFisher Scientific, Paisley, UK, catalog number: C36995) on a Accuri C6 Flow Cytometer (Becton Dickinson, Oxford, UK). To reflect physiological conditions, the PBMC fraction was diluted to match the monocyte concentration in whole blood for each participant (≈0.2 to 1.0 × 10^6^/mL). PBMCs were then added in duplicate to fibronectin‐coated polyester (PET) inserts (5 μm pore size, Merck Life Sciences, Gillingham, UK, catalog number: MCMP06H48) and placed into 6‐well non‐adhesive plates (ThermoFisher Scientific, Paisley, UK, catalog number: 250239) containing 3 mL of 50% cultured media from pre‐adipocytes (Caltag Medsystems, Buckingham, UK, catalog number: CM‐A10) or RPMI (migration control, ThermoFisher Scientific, Paisley, UK, catalog number: 21875091). The dominant chemokines in the cultured media were CCL2, CCL5, CCL7, CCL8, and CX3CL1. The 6‐well plate was then placed on a two‐dimensional orbital shaker at 77 rpm within an incubator at 37° and 5% CO_2_ for 3 h, simulating physiological flow and conditions within the circulation (3.2 dyn/cm^3^) (Buchanan et al., 2014). The concentration of cultured media and incubation times were determined from in‐house validation experiments from our laboratory (Wadley et al., 2021).

After 3 h, non‐adherent PBMCs were removed from the upper side of the PET insert, and then the upper side of the PET insert was washed twice with 1 mL of D‐PBS (ThermoFisher Scientific, Paisley, UK, catalog number: 14190169). Then, 1 mL of enzyme free, EDTA‐based dissociation media (Merck Life Sciences UK Ltd, Gillingham, UK, catalog number: S‐014‐B) was added to the upper side of the insert (4°, 30 min), followed by five washes with 1 mL D‐PBS to remove and collect the adhered PBMCs. The underside of the PET insert and the wells of the non‐adhesive plates were treated identically to collect PBMCs that had migrated through the PET insert. Adhered and migrated cells were collected into separate tubes and then washed in D‐PBS ready for subsequent counting and phenotyping using flow cytometry.

### 2.5 Flow cytometry

Pre‐migration (baseline) adhered and migrated PBMCs were counted using CountBright™ beads as stated above. For each (baseline, adhered, and migrated) sample, 1.75 × 10^5^ PBMCs were stained using fluorescently conjugated antibodies for identification of monocyte subsets and chemokine receptor expression (CCR2 and CCR5). Cells were incubated with CD14‐FITC, CD16‐PE, CCR2‐Alexa Flour‐647, CCR5‐APC antibodies, and 7‐AAD (Becton Dickinson, Oxford, UK, catalog numbers: 555397, 555407, 558406, 560748, 559925, respectively) at 4°C for 30 min in the dark. PBMCs were then washed twice with FACS buffer for 5 min at 300×*g*. Single‐stained controls were used weekly to adjust the compensation. Gates were established using fluorescence minus one controls.

**FIGURE 1:**
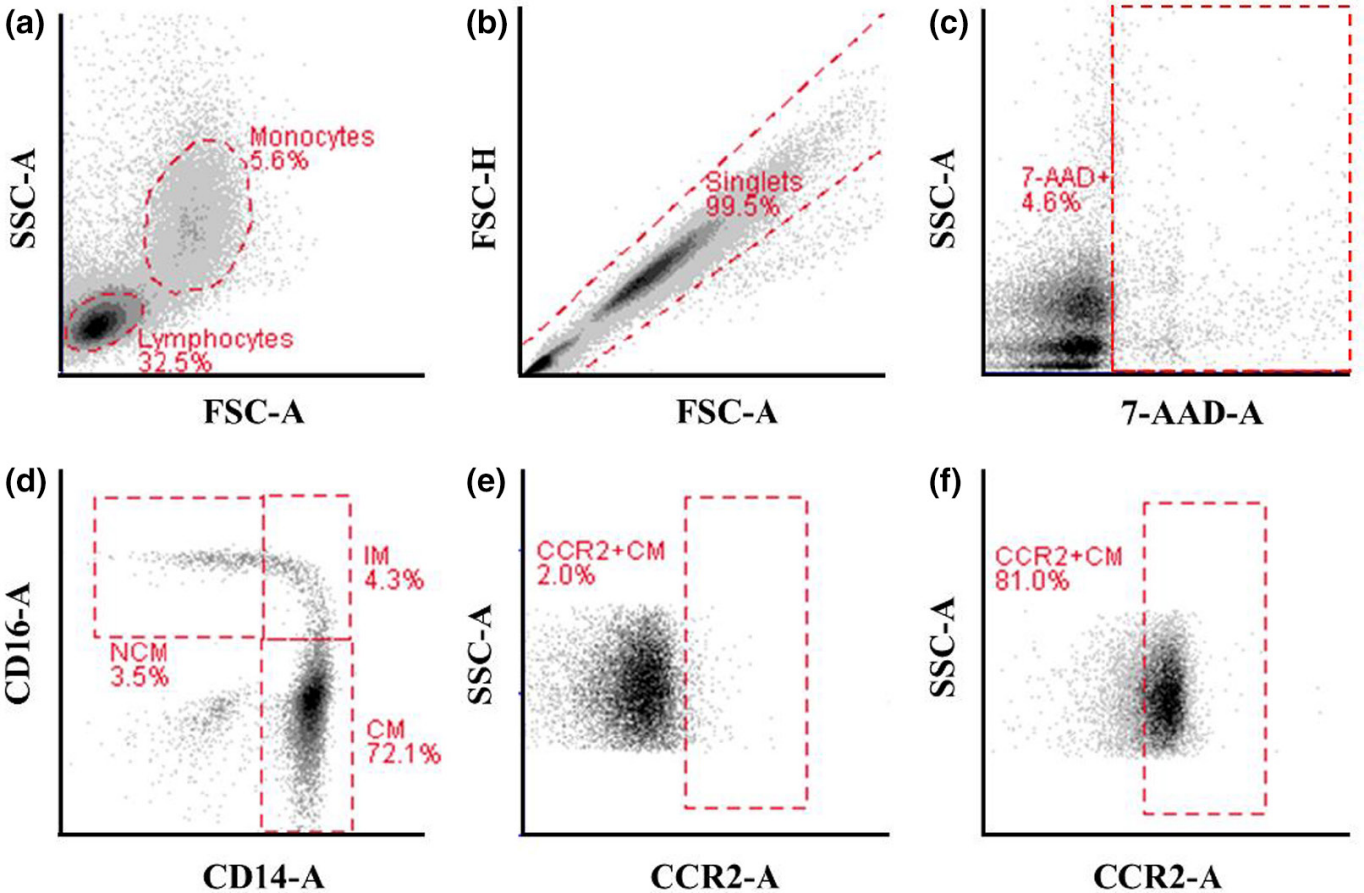
Gating strategy for monocyte subsets and their respective chemokine receptor expression. (a) Monocytes gated on forward light scatter (FSC) versus side light scatter (SSC). (b) Doublets were excluded using FSC area versus FSC height. (c) Left, non‐viable cells excluded using 7‐AAD. (d) Monocyte subsets were identified using CD‐14 area versus CD‐16 area excluding doublets and 7‐AAD+ cells. (e) The positive gate for CCR2+ monocytes was determined using a fluorescence minus one control. (f) CCR2+ monocytes determined on CCR2+ area versus SSC area. 7‐AAD, 7‐aminoactinomycin D; CM, classical monocytes; CCR2+, C‐C chemokine receptor‐2+; IM, intermediate monocytes; NCM, non‐classical monocytes.

BD C6 Accuri software (Becton Dickinson, Oxford, UK) was used to analyze flow cytometry data (Figure 1). Monocytes were gated on forward versus side scatter. Doublets were excluded using FSC‐A versus FCS‐H plots. Non‐viable (e.g., 7‐AAD+) cells were excluded. CD14+ and CD16+ plots were used to determine CM (CD14++CD16‐), IM (CD14++CD16+), and NCM (CD14+CD16++) proportions (Figueroa‐Vega et al., 2020). Histogram plots of the cells in the total and CM regions that positively expressed CCR2 and CCR5 were used to calculate the proportion of chemokine receptor positive cells. Mean fluorescent intensity—used to determine the density of chemokine receptor expression—was also determined for total monocyte and CM populations. The percentage of monocyte subsets and CCR2+ and CCR5+ monocytes were used with whole blood cell counts to determine the circulating number of chemokine receptor positive monocytes for each population.

### 2.6 Statistical analyses

Data were analyzed using the statistical package for social sciences (SPSS version 27). Model residuals for outcomes were explored using histograms. Normally distributed data are presented as the arithmetic mean (SD) and pairwise comparisons are based on the mean differences and 95% confidence intervals (95% CI) of the mean absolute difference. Skewed data were natural log transformed prior to analysis and analysis was performed on the log‐arithmetic transformation of the data, but then back‐transformed for presentation (Bland & Altman, 1996a). Skewed data are highlighted in Figure legends. Statistical significance was accepted as *p* < 0.05. Effect sizes (ES) are presented to supplement findings. An effect size of 0.2 was considered the minimum value for a meaningful difference (Cohen, 1988).

Participant characteristics were compared using linear models with ethnicity (SA vs. WE) and central obesity status (central obesity vs. lean) as fixed factors. Blood and ex vivo migration variables were compared as above but included ethnicity‐by‐central obesity status as an interaction term. Migration and adhesion variables are reported as absolute and normalized for differences in number of circulating monocytes between participants (relative). The interaction was incorporated to investigate whether central obesity impacted the migration and adhesion of monocytes to a greater extent in SAs compared with WEs. Pearson's correlation (normally distributed data) or Spearman's correlation (non‐normally distributed data) was performed to identify any correlations between cardiometabolic markers and monocyte outcomes (counts, absolute migration and adhesion, and relative migration and adhesion).

## 3 RESULTS

### 3.1 Participant characteristics, metabolic markers, and blood pressure

Insulin, HOMA‐IR, and CRP were significantly higher in SAs versus WEs (ES ≥ 0.65) (Table 1). There were no significant ethnic differences for other outcomes (ES ≤ 0.62) (Table 1). Participants with central obesity had a higher BMI, waist circumference, hip circumference, waist‐to‐hip ratio, body fat percentage, circulating glucose, TAG, insulin, HOMA‐IR, and DBP than participants who were lean (ES ≥ 0.71) (Table 1). Obesity status did not significantly affect other outcomes (ES ≤ 0.52) (Table 1).

**TABLE 1:** Participant characteristics, fasting metabolic markers, resting blood pressure, and monocyte subset concentrations.

Variable	Group mean (SD or 95% CI)				Main effects (95% CI)	
	LE‐WE (*n* = 10)	CO‐WE (*n* = 10)	LE‐SA (*n* = 10)	CO‐SA (*n* = 10)	Ethnicity (SA vs. WE)	Obesity (CO vs. LE)
Age (years)	32 (7)	36 (8)	36 (7)	36 (13)	2.0 (−4 to 8)	2.7 (−3 to 9)
Body mass index (kg/m^2^)	23.9 (1.8)	29.1 (2.0)	24.5 (1.5)	31.5 (5.0)	1.5 (−0.4 to 3.4)	6.1 (4.2 to 8.0)*
Waist circumference (cm)	79.6 (4.7)	101.1 (5.6)	83.2 (3.4)	103.1 (5.9)	2.8 (−0.4 to 6.0)	20.7 (17.5 to 23.9)*
Hip circumference (cm)	98.5 (3.6)	104.8 (5.4)	98.3 (1.8)	108.7 (9.0)	1.9 (−1.7 to 5.5)	8.4 (4.8 to 12.0)*
Waist‐to‐hip ratio	0.81 (0.04)	0.97 (0.05)	0.85 (0.05)	0.95 (0.06)	0.01 (−0.02 to 0.05)	0.13 (0.10 to 0.16)*
Body fat (%)^a^	18.8 (4.5)	31.4 (4.5)	21.4 (5.4)	32.4 (4.9)	1.8 (−1.3 to 4.9)	11.8 (8.7 to 14.9)*
TC (mmol/L)	4.4 (1.5)	4.8 (0.7)	4.9 (1.0)	5.4 (1.5)	0.5 (−0.3 to 1.3)	0.4 (−0.4 to 1.1)
HDL (mmol/L)	1.2 (0.3)	1.2 (0.3)	1.3 (0.4)	1.0 (0.2)	−0.1 (−0.3 to 0.1)	−0.2 (−0.4 to 0.0)
LDL (mmol/L)	2.4 (0.9)	2.9 (0.7)	3.0 (0.8)	3.3 (1.2)	0.5 (−0.1 to 1.1)	0.4 (−0.2 to 1.0)
Glucose (mmol/L)	4.7 (0.4)	5.3 (0.2)	5.1 (0.2)	5.2 (0.3)	0.1 (−0.1 to 0.3)	0.3 (0.1 to 0.5)*
TAG (mmol/L)	1.2 (1.2)	1.3 (0.5)	0.9 (0.3)	2.1 (1.0)	0.2 (−0.3 to 0.7)	0.6 (0.1 to 1.2)*
Insulin (pmol/L)	28.1 (6.0)	58.7 (15.2)	43.2 (22.0)	83.1 (41.9)	19.7 (3.7 to 35.8)*	35.2 (19.2 to 51.3)*
HOMA‐IR	0.99 (0.26)	2.30 (0.66)	1.62 (0.81)	3.23 (1.69)	0.78 (0.13 to 1.42)*	1.46 (0.82 to 2.10)*
TNF‐α (pg/mL)	8.3 (2.0)	8.3 (0.7)	9.0 (1.3)	10.2 (3.4)	1.3 (−0.1 to 2.6)	0.6 (−0.7 to 2.0)
CRP (mg/L)	0.3 (0.2–0.6)	0.9 (0.5–1.7)	0.9 (0.5–1.8)	1.8 (0.9 to 3.5)	1.4 (0.1 to 2.7)*	1.1 (−0.2 to 2.4)
SBP (mmHg)	126 (4)	122 (4)	123 (8)	125 (8)	0 (−4 to 4)	−1 (−5 to 3)
DBP (mmHg)	73 (69–77)	83 (79–88)	82 (78 to 87)	81 (77–86)	4 (−0 to 8)	4 (0 to 8)*
Monocyte count (cells/μL)	0.39 (0.07)	0.43 (0.08)	0.46 (0.12)	0.52 (0.03)	0.49 (0.45 to 0.52)*	0.05 (0.00 to 0.10)*
CM Conc. (cells/μL)	255.6 (49.1)	297.3 (65.9)	323.1 (84.9)	376.6 (33.3)	73.4 (33.9 to 112.7)*	47.6 (8.2 to 88.9)*
IM Conc. (cells/μL)	18.7 (8.7)	25.1 (8.2)	25.7 (11.9)	35.8 (6.3)	8.7 (2.9 to 14.5)*	8.3 (2.5 to 14.1)*
NCM Conc. (cells/μL)	24.0 (7.4)	33.5 (15.4)	33.3 (13.4)	44.3 (13.0)	9.9 (1.8 to 18.0)*	10.3 (2.2 to 18.4)*
CCR2+ Monocyte Conc. (cells/μL)	227.6 (50.6)	277.3 (77.7)	299.5 (76.5)	384.3 (49.7)	89.5 (47.7 to 131.2)*	67.3 (25.5 to 109.0)*
CCR2+ CM Conc (cells/μL)	220.5 (39.3)	248.0 (49.5)	258.0 (73.6)	306.6 (32.1)	48.2 (15.3 to 81.0)*	38.1 (5.2 to 70.9)*
CCR5+ Monocyte Conc. (cells/μL)	9.4 (3.5)	12.3 (11.0)	18.2 (5.3)	32.7 (5.7)	14.8 (10.2 to 19.3)*	8.9 (4.3 to 13.4)*

*Note*: Data were analyzed using mixed models with ethnicity and central obesity as fixed factors. Values are mean and standard deviations for normally distributed data and mean (95% CI) for skewed data.

Abbreviations: 95% CI, 95% confidence interval; CCR, C‐C chemokine receptor; CM, classical monocytes; CO‐SA, South Asian men with central obesity; CO‐WE, white European men with central obesity; CRP, C‐reactive protein; DBP, diastolic blood pressure; HDL, high‐density lipoprotein cholesterol; HOMA‐IR, homeostatic model assessment for insulin resistance; IM, intermediate monocytes; LDL, low‐density lipoprotein cholesterol; LE‐SA, lean South Asians; LE‐WE, lean white Europeans; NCM, non‐classical monocytes; SBP, systolic blood pressure; SD, standard deviation; TAG, triacylglycerol; TC, total cholesterol; TNF‐α, tumor necrosis factor‐alpha.

* Main effect of group (*p* ≤ 0.047).

^a^ Body fat percentage determined by bioelectrical impedance.

### 3.2 Monocyte phenotype in blood

Total monocytes, CM, IM, NCM, CCR2+M, CCR2+CM, and CCR5+ monocytes were higher for SA compared with WE participants (ES ≥ 0.74) (Table 1). Participants with central obesity also had higher concentrations of all monocyte subsets compared with participants who were lean (ES ≥ 0.59) (Table 1).

### 3.3 Absolute and relative ex vivo migration

**FIGURE 2:**
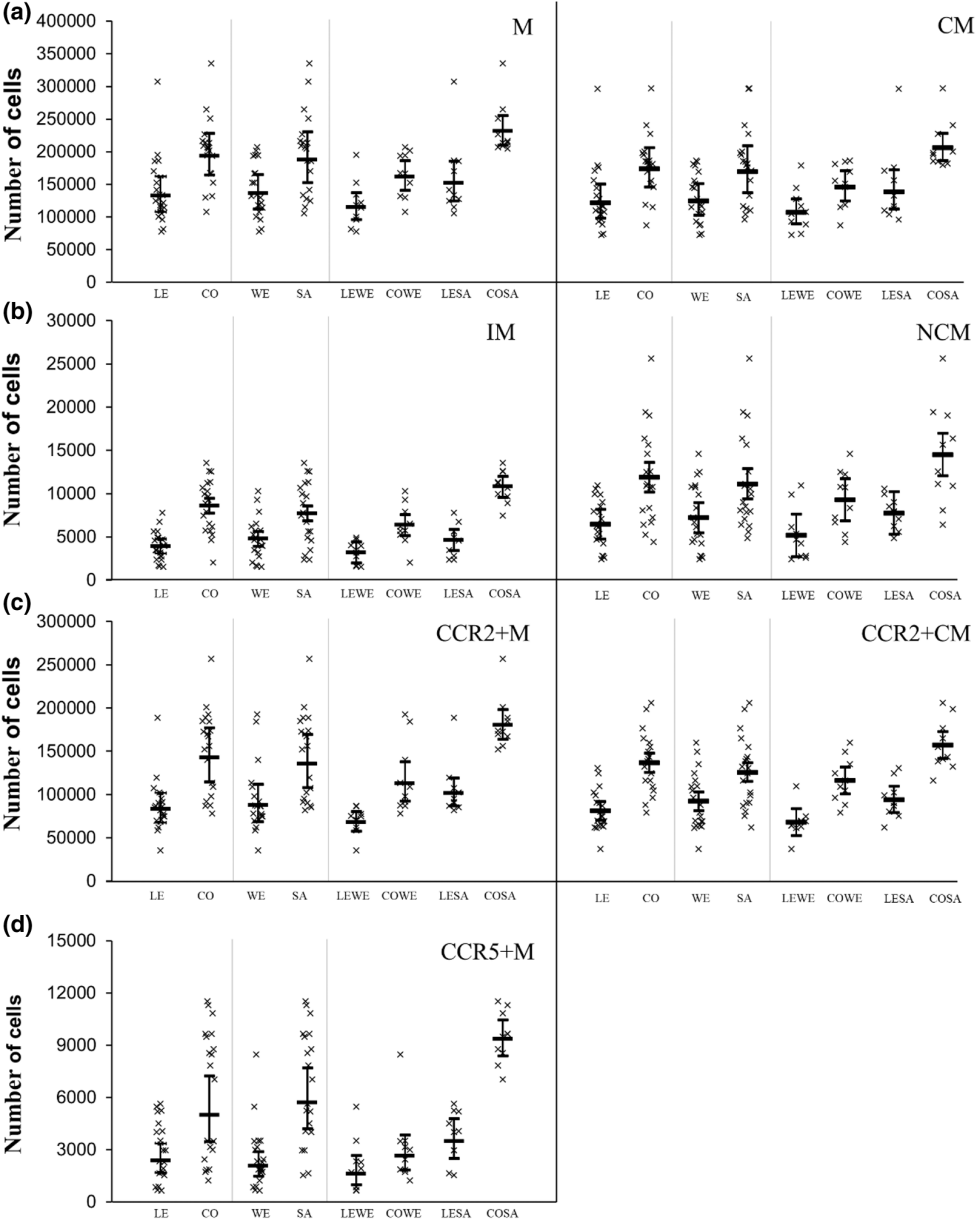
Absolute migration between participants who were lean (LE) and had central obesity (CO), white Europeans (WE) and South Asians (SA), and white Europeans and SAs with and without central obesity (LE‐WE, CO‐WE, LE‐SA, CO‐SA). Skewed data were natural log transformed prior to analysis and analysis was performed on the log‐arithmetic transformation of the data, but then back‐transformed for presentation. This was performed for M, CM, NCM, CCR2+M, and CCR5+M. Data presented as individual data points (95% confidence intervals). Main effects of ethnicity, central obesity status, and ethnicity‐by‐central obesity status interactions are presented in text. CCR, C‐C chemokine receptor; CM, classical monocytes; IM, intermediate monocytes; M, monocytes; NCM, non‐classical monocytes.

**FIGURE 3:**
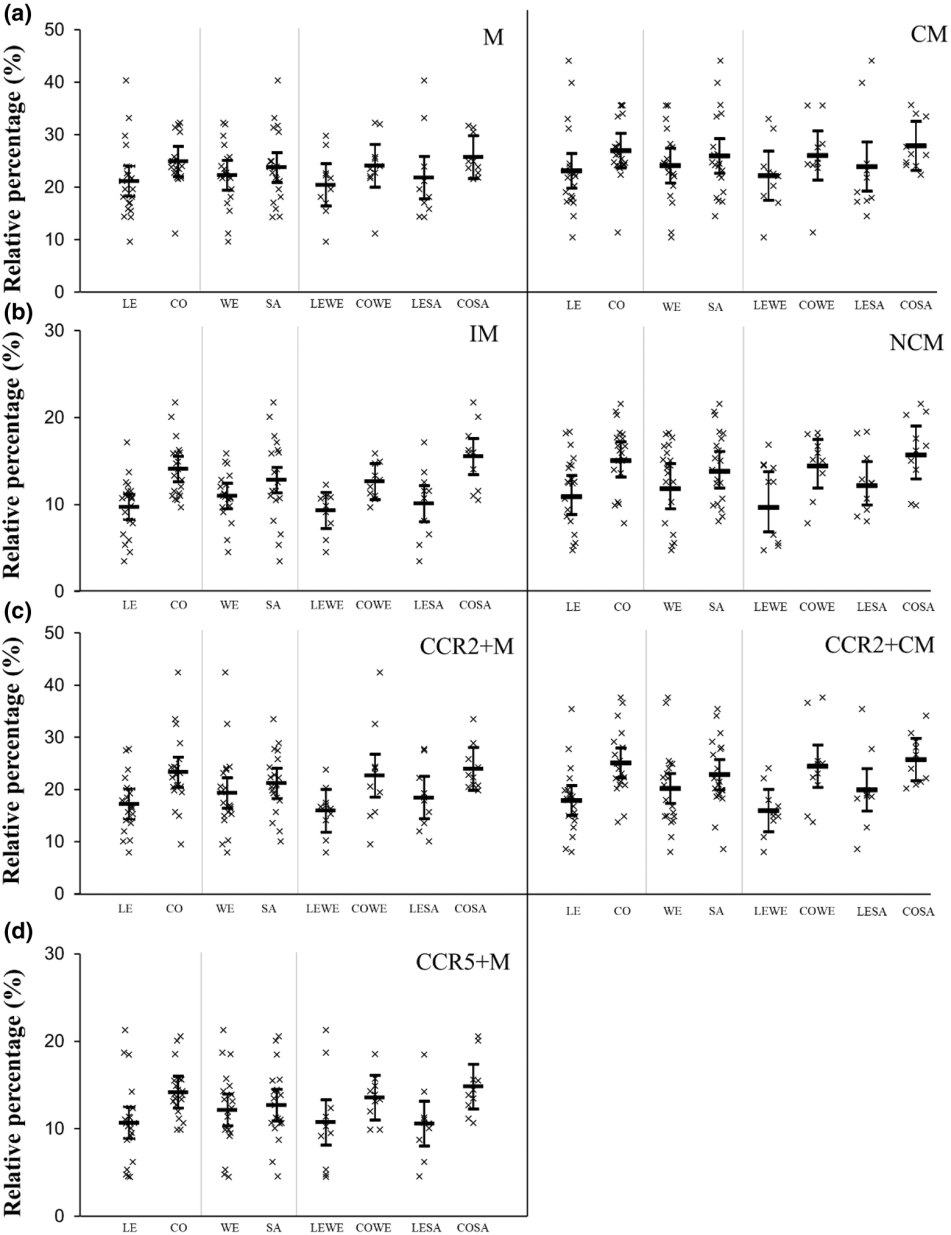
Relative migration between participants who were lean (LE) and had central obesity (CO), white Europeans (WE) and South Asians (SA), and white Europeans and SAs with and without central obesity (LE‐WE, CO‐WE, LE‐SA, CO‐SA). Data presented as individual data points (95% confidence intervals). Main effects of ethnicity, central obesity status, and ethnicity‐by‐central obesity status interactions are presented in text. CCR, C‐C chemokine receptor; CM, classical monocytes; IM, intermediate monocytes; M, monocytes; NCM, non‐classical monocytes.

Absolute and relative changes in ex vivo monocyte migration are presented in Figures 2 and 3, respectively. Absolute migration of all monocyte subsets was higher for SAs compared to WEs (*p* ≤ 0.003; ES ≥ 0.83) and for participants with central obesity compared with participants who were lean (*p* < 0.001; ES ≥ 1.04). Ethnicity‐by‐central obesity status interactions revealed an interaction for IM (*p* = 0.017) only. The magnitude of migration for IM was higher in CO‐SA versus LE‐SA (6159 higher, 95% CI, 4452–7865) compared with CO‐WE versus LE‐WE (3168 higher, 95% CI, 1462–4875).

For relative migration, there was no main effect of ethnicity for any of the monocyte subsets (*p* ≥ 0.086; ES ≤ 0.46), indicating that monocyte migration is higher in SAs due to higher numbers of circulating of cells. The relative migration of IM, NCM, CCR2+M, CCR2+CM, and CCR5+M was higher in participants with central obesity compared with participants who were lean (*p* ≤ 0.009; ES ≥ 0.90), but there was no main effect on the relative migration of M or CM (*p* ≥ 0.067; ES ≤ 0.61). There were no ethnicity‐by‐central obesity interactions for any of the monocyte subsets (*p* ≥ 0.314).

### 3.4 Absolute and relative ex vivo adhesion

**FIGURE 4:**
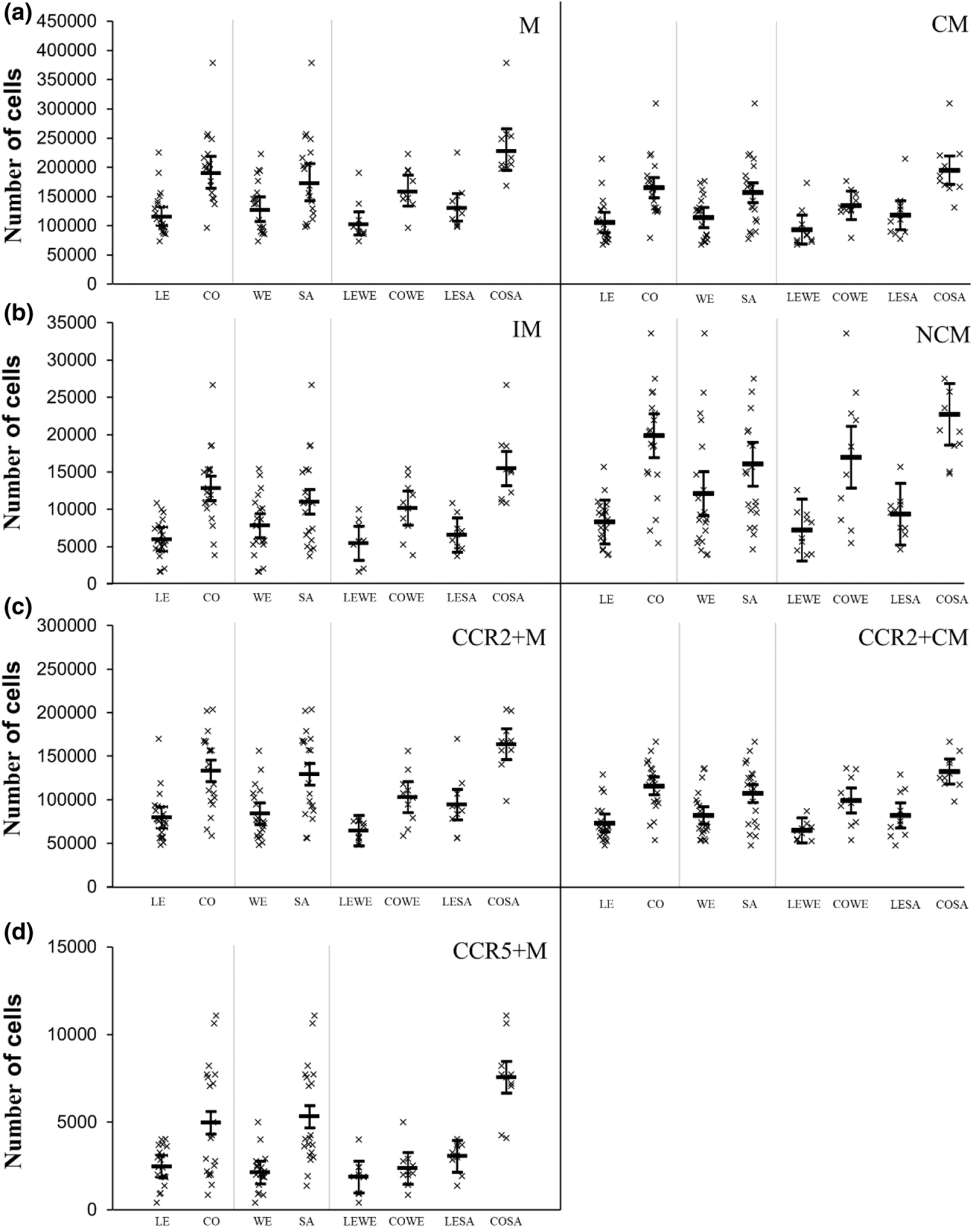
Absolute adhesion between participants who were lean (LE) and had central obesity (CO), white Europeans (WE) and South Asians (SA), and white Europeans and SAs with and without central obesity (LE‐WE, CO‐WE, LE‐SA, CO‐SA). Skewed data were natural log transformed prior to analysis and analysis was performed on the log‐arithmetic transformation of the data, but then back‐transformed for presentation. This was performed for M, CM, and IM. Data presented as individual data points (95% confidence intervals). Main effects of ethnicity, central obesity status, and ethnicity‐by‐central obesity status interactions are presented in text. CCR, C‐C chemokine receptor; CM, classical monocytes; IM, intermediate monocytes; M, monocytes; NCM, non‐classical monocytes.

**FIGURE 5:**
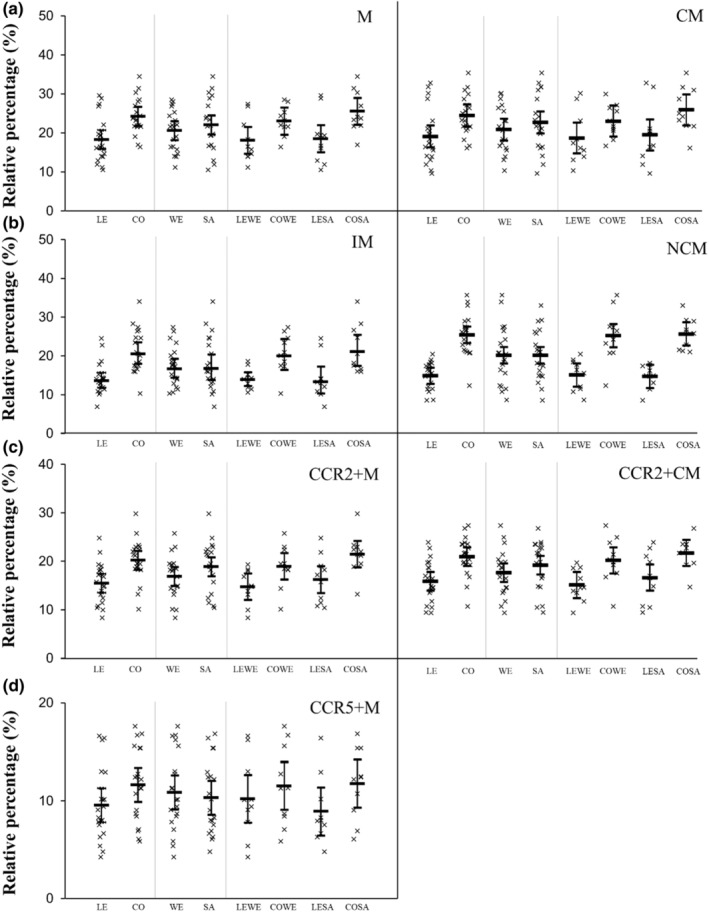
Relative adhesion between participants who were lean (LE) and had central obesity (CO), white Europeans (WE) and South Asians (SA), and white Europeans and SAs with and without central obesity (LE‐WE, CO‐WE, LE‐SA, CO‐SA). Skewed data were natural log transformed prior to analysis and analysis was performed on the log‐arithmetic transformation of the data, but then back‐transformed for presentation. This was performed for IM. Data presented as individual data points (95% confidence intervals). Main effects of ethnicity, central obesity status, and ethnicity‐by‐central obesity status interactions are presented in text. CCR, C‐C chemokine receptor; CM, classical monocytes; IM, intermediate monocytes; M, monocytes; NCM, non‐classical monocytes.

Absolute and relative ex vivo monocyte adhesion is presented in Figures 4 and 5, respectively. Absolute adhesion of all monocyte subsets was higher in SAs than WEs (*p* ≤ 0.008; ES ≥ 0.63) apart from NCM (*p* = 0.061; ES = 0.45). Participants with central obesity had higher absolute adhesion of all monocyte subsets compared with people who were lean (*p* < 0.001; ES ≥ 1.05). Ethnicity‐by‐central obesity status interactions revealed an interaction for CCR5+M (*p* = <0.001), but not for any of the other monocyte subsets (*p* ≥ 0.072). The magnitude of adhesion for CCR5+M was higher in CO‐SA versus LE‐SA (4493 higher, 95% CI, 3211–5775) compared with CO‐WEs versus LE‐WE (494 higher, 95% CI, −787 to 1776).

For relative adhesion, there was no main effect of ethnicity for any of the monocyte subsets (*p* ≥ 0.155; ES ≤ 0.41). Participants with central obesity had higher relative adhesion of all monocyte subsets compared with participants who were lean (*p* ≤ 0.009; ES ≥ 0.88) apart from CCR5+M (*p* = 0.094; ES = 0.55). There were no ethnicity by central obesity interactions for any of the monocyte subsets (*p* ≥ 0.548).

### 3.5 Chemokine receptor expression

Expression of CCR2 and CCR5 on total monocytes at baseline and at stages of the ex vivo assay (adhered and migrated) is presented in Table 2. A main effect of ethnicity revealed higher expression of CCR2 on total monocytes for SAs compared to WEs at all stages of the migration assay (*p* ≤ 0.038; ES ≥ 0.59), and CCR5 on migrated monocytes (*p* = 0.007; ES = 0.78). A main effect of central obesity status revealed higher expression of CCR2 and CCR5 on total monocytes for all stages in participants with central obesity compared to participants who were lean (*p* ≤ 0.045, ES ≥ 0.66). There were no ethnicity‐by‐central obesity interactions (*p* ≥ 0.305).

**TABLE 2:** Receptor expression. Expression in monocytes isolated at baseline, after adhesion, and after migration are indicated for CCR2 and CCR5.

Variable	Group mean (95% CI)				Main effects (95% CI)	
	LE‐WE (*n* = 10)	CO‐WE (*n* = 10)	LE‐SA (*n* = 10)	CO‐SA (*n* = 10)	Ethnicity (SA vs. WE)	Obesity (CO vs. LE)
CCR2						
Baseline	4810 (4249–5369)	5432 (4872–5992)	5417 (4857–5977)	6012 (5451–6571)	594 (33–1154)*	608 (48–1169)*
Adhered	4132 (3681–4583)	5072 (4621–5523)	4668 (4217–5119)	5506 (5055–5957)	485 (34–936)*	889 (438–1340)*
Migrated	4102 (3620–4584)	5085 (4603–5567)	4748 (4265–5230)	5589 (5106–6071)	574 (92–1056)*	912 (429–1394)*
CCR5						
Baseline	3849 (3096–4601)	4698 (3946–5451)	4445 (3693–5198)	5133 (4381–5886)	515 (−236 to 1268)	768 (16–1521)*
Adhered	3576 (2908–4244)	4511 (3843–5179)	4342 (3674–5010)	5021 (4353–5689)	638 (−30 to 1305)	806 (138–1474)*
Migrated	3561 (3128–3994)	4591 (4157–5024)	4395 (3961–4828)	4980 (4547–5414)	611 (178–1044)*	807 (374–1241)*

*Note*: Data were analyzed using mixed models with ethnicity (LE‐SA and CO‐SA, LE‐WE and CO‐WE grouped) and central obesity (LE‐WE and LE‐SA, CO‐WE and CO‐SA grouped) as fixed factors.

Abbreviations: CCR, C‐C chemokine receptor; CO‐SA, South Asian men with central obesity; CO‐WE, white European men with central obesity; LE‐SA, lean South Asians; LE‐WE, lean white Europeans.

* Main effect of group (*p* ≤ 0.045). There were no ethnicity‐by‐central obesity interactions (*p* ≥ 0.305).

## 4 DISCUSSION

In line with the hypothesis, principal findings show (1) Absolute, but not relative, adhesion and migration of monocytes is higher in SAs than WEs (2) central obesity is associated with higher absolute and relative migration and adhesion of monocytes (3) there are limited ethnicity‐by‐central obesity interactions, suggesting central obesity heightens the capacity for adhesion and migration of monocytes to a similar extent in SAs and WEs. The results also indicate that SAs have a higher concentration of all monocyte subsets compared with WEs, and, importantly, SAs who are lean have similar migration and adhesion of monocyte subsets to WEs with central obesity. An interesting interaction for absolute IM migration was also highlighted, with central obesity elevating the absolute migration of IM to a greater extent in SAs compared with WEs.

The higher concentration of all monocyte subsets in SAs compared with WEs highlights a disturbance in immune profiles in SAs which is a source of greater inflammation (Central Illusration). This may be one of the reasons for a greater risk of developing inflammatory diseases such as diabetes and CVD in SAs compared with WEs (Sattar & Gill, 2015). Numbers of CM (CD14++CD16‐) were ~27% higher in SAs compared with WEs. CM are more able to migrate to cues stemming from injured or inflamed tissues and are characterized by their secretion of pro‐inflammatory proteins such as IL‐6 and IL‐8, the ability to differentiate into monocyte‐derived macrophages and dendritic cells, and their role in tissue inflammation (Kapellos et al., 2019). Numbers of IM (CD14++CD16+) were ~41% higher in SAs than WEs. IM are more involved with vascular inflammation and are characterized by their high expression of CCR5 (Kapellos et al., 2019).

CCR5 is a receptor for CCL5/RANTES ligands, inducing monocyte migration (Cross et al., 1997). Under homeostatic conditions, CCR5 is not overly expressed. However, it has been demonstrated that CCR5 can become highly expressed during inflammation as CCR5+ monocytes migrate from the bone marrow to the circulation and then to the site of infection/inflammation where they are involved in the control of local inflammation and pathogen clearance through the recruitment of T cells (Castanheira et al., 2019). The population of IM expands in people who need a rapid defense against pathogens, such as in the blood of patients with sepsis (Kapellos et al., 2019). When stimulated, IM release TNF‐α, IL‐6, and IL‐1β, contributing to vascular inflammation (Kapellos et al., 2019). Consequently, numbers of CCR5+ monocytes are positively associated with accumulation of atherosclerotic plaques (Tacke et al., 2007). In the current study, higher numbers of IM in SAs indicate a heightened level of systemic inflammation. Concentrations of NCM (CD14+CD16++) were also ~35% higher in SAs and have similar function to IM but produce more TNF‐α and IL‐1β when stimulated (Wong et al., 2011).

Collectively, this perturbation in monocyte biology may explain the more pro‐inflammatory phenotype in SAs compared with WEs, with studies reporting higher CRP and IL‐6 concentrations in healthy middle‐aged SA men (Chambers et al., 2001), women (Peters et al., 2013), and men with central obesity (Roberts et al., 2023). This is supported by the current findings with 1.4 mg/L higher CRP and 1.3 pg/mL higher TNF‐α concentrations in SAs compared with WEs. Interestingly, concentrations of TNF‐α were similar between WEs with central obesity and SAs who were lean, as were numbers of IM and NCM. This does suggest that differences in the number of IM and NCM—which are a major source of TNF‐α—may drive adverse inflammatory profiles seen in SAs (Chambers et al., 2001; Peters et al., 2013; Roberts et al., 2023). Furthermore, CRP concentrations were similar between WEs with central obesity and SAs who were lean. This suggests similar levels of inflammation in WEs with central obesity compared to lean SAs, which provides new insight into mechanisms contributing to the elevated CVD risk in SAs (Ahmed & El‐Menyar, 2015).

This is also supported by the metabolic data. In the current study, SAs had higher HOMA‐IR values than WEs, supporting cluster‐based phenotypic analyses demonstrating a higher frequency of insulin resistance in SA populations compared to WEs (Ke et al., 2022). This is typically due to a limited innate capacity to secrete insulin meaning even a small reduction in insulin secretion through aging or β‐cell exhaustion results in type 2 diabetes mellitus (Ke et al., 2022). The higher HOMA‐IR in our SA cohort was not surprising as the participants were in their late 30s and HOMA‐IR is especially high in SAs compared to WEs between the ages of 40–60 (Ke et al., 2022). Although SA participants had significantly higher HOMA‐IR than WEs—suggesting a heightened risk of diabetes—there were no significant differences in CVD risk factors such as TAG, glucose, blood pressure, or types of cholesterol. Collectively, the immune, inflammatory, and metabolic data provide new insight into the elevated CVD risk in SAs by suggesting heightened inflammation and adverse immune profiles play a pivotal role. Central obesity was also associated with higher numbers of monocyte and higher concentrations of cardiovascular and inflammatory markers, supporting our earlier work (Wadley et al., 2021).

The above provides a good snapshot of immune profiles in the blood of SAs compared with WEs; however, the movement of monocytes is fundamental in chronic inflammation. The damaging effects of excessive transmigration and adhesion of the monocyte subsets are not limited to local impacts. For example, monocytes produce IL‐6 which promotes leukocyte recruitment from the circulation into larger (e.g., liver tissue), and smaller (e.g., atherosclerotic plaques) sites of inflammation by upregulating cell expression of adhesion molecules and chemokines (Bishop et al., 2022). Monocyte‐derived inflammatory cytokines also inhibit insulin signaling, impede mitochondrial dynamics, and promote reactive oxygen species production and subsequent cell death, contributing to systemic inflammation (Bishop et al., 2022). Furthermore, persistent diapedesis stimulates bone marrow hematopoiesis increasing circulating numbers of monocytes (Idzkowska et al., 2015). In the present study, the absolute adhesion and migration of monocyte subsets was higher in SAs compared to WEs. However, relative migration and adhesion were similar.

This has physiological significance. Although there is a similar percentage of cells migrating and adhering, the absolute number is still greater in SAs. Heightened migration is associated with macrophage accumulation in the tissue, a pro‐inflammatory microenvironment in the tissue, and higher pro‐inflammatory cytokine secretion (Bishop et al., 2022). This is supported by the current data, with higher CRP and TNF‐α concentrations in SAs compared with WEs. This again provides new insight into the elevated CVD risk in SAs. SAs have an adverse immune profile, heightened absolute immune cell adhesion to the vascular wall, and higher immune cell migration to adipose tissue. Over time, this will contribute to vascular and tissue inflammation, contributing to CVD (Bishop et al., 2022). Therefore, considering relative immune cell adhesion and migration are similar between SAs and WEs, therapies and/or pharmaceuticals should focus on lowering immune cell counts and baseline inflammatory profiles in SAs to mitigate the contribution of heightened immune cell adhesion and migration to CVD (Bishop et al., 2022).

The higher migration and adhesion of inflammatory monocytes may be driven by higher expression of CCR2 and CCR5, both of which were higher in SAs compared with WEs in the present study and have previously been shown to positively correlate with insulin insensitivity on total monocytes (Blanks et al., 2020). Furthermore, there were a greater number of CCR2+M, CCR2+CM, and CCR5+M migrating and adhering in SAs compared to WEs. Our data also show CCR2 and CCR5 expression was lower after ex vivo adhesion and migration suggesting receptor internalization drives monocyte transmigration. The chemokine receptors have high affinity for CCL2 (MCP‐1) and CCL5 (RANTES), which are released from cells or tissues under metabolic and/or inflammatory stress (Wadley et al., 2021) and were present in the adipose‐conditioned media used here.

**FIGURE 6:**
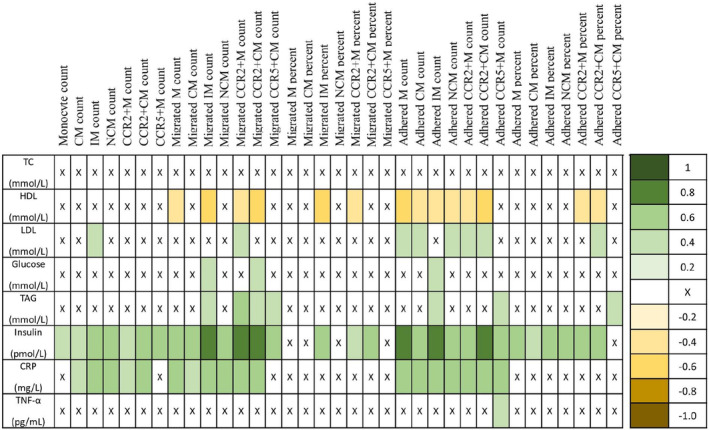
Associations between cardiometabolic markers and monocyte outcomes (counts, absolute migration and adhesion, and relative migration and adhesion). A heat map illustrating correlations. Strength of associations are given in the colored scale bar. X means non‐significant. CCR, C‐C chemokine receptor; CM, classical monocytes; CRP, C‐reactive protein; HDL, high‐density lipoprotein cholesterol; IM, intermediate monocytes; LDL, low‐density lipoprotein cholesterol; NCM, non‐classical monocytes; TAG, triacylglycerol; TC, total cholesterol; TNF‐α, tumor necrosis factor‐alpha.

We further interrogated our data by looking at associations between monocyte counts, migration, and adhesion with cardiometabolic markers. The present study provides novel evidence that a negative correlation exists between HDL concentrations and the absolute migration of M, IM, CCR2+M, CCR2+CM, the relative migration of IM and CCR2+M, the absolute adhesion of all monocyte subsets apart from CCR5+M, and the relative adhesion of NCM, CCR2+M, and CCR2+CM (Figure 6). Additionally, absolute adhesion of all monocyte subsets apart from IM and CCR5+M was positively correlated with LDL concentrations. Devêvre et al. (2015) identified a negative correlation between CCR2 and HDL, whereas CCR5 was positively linked to obesity‐related metabolic traits (Devêvre et al., 2015). While the previous research focused on absolute numbers, the present results demonstrate that the absolute number of monocytes that adhere to the vascular wall may influence abnormal lipid profiles. Our data also show a positive correlation between numbers of monocyte subsets, absolute migration, and absolute and relative adhesion with insulin and CRP concentrations (Figure 6). Insulin and CRP are established clinical markers of health (Pradhan et al., 2003). The pro‐inflammatory cytokines produced by monocytes can induce widespread insulin resistance by inhibiting insulin signaling and impairing lipid metabolism in liver and skeletal muscle tissue (Kojta et al., 2020). This may also be related to the inhibition in the synthesis of adiponectin—an insulin sensitizing and anti‐inflammatory protein—by pro‐inflammatory cytokines such as IL‐6, exacerbating the pro‐inflammatory environment in those with high monocyte counts, and high monocyte migration and adhesion (Ellulu et al., 2017). Consequently, the association between monocyte biology and cardiovascular profiles presented here provides new insight as to why SAs have a higher risk of CVD than WEs, even after accounting for differences in body fat distribution (Alenaini et al., 2020).

Although ethnicity‐by‐obesity interactions were limited, central obesity elevated the absolute migration of IM, and the absolute adhesion of CCR5+M, to a greater extent in SAs compared to WEs. As IM are involved in vascular inflammation, the higher migratory capacity of IMs in SAs with central obesity provides new insight as to why SAs with central obesity have adverse endothelial function compared to WEs with central obesity who are matched for age (Roberts et al., 2023). CCR5+M are related to neuroinflammation (Ubogu et al., 2006) and insulin insensitivity (Blanks et al., 2020), therefore the greater adhesion capacity of CCR5+M in SAs with central obesity provides insight as to why SAs have higher type 2 diabetes risk compared with WEs for any given BMI (Sattar & Gill, 2015).

## 5 LIMITATIONS

The strengths of the study include utilizing a dynamic migration model that simulates physiologic blood flow, coupled with flow cytometry and the use of adipose‐specific media. SA populations are heterogenous between and within SA countries. SA research is normally dominated by an Indian cohort due to India dominating the population of South Asia and populous Indian regions in the West. Although the SA sample size was 20 in this study, the study involved participants with ethnic heritage to Pakistan, Bangladesh, Bhutan, Nepal, and India. The sample size is similar to other laboratory mechanistic studies in SA populations and concerns about heterogeneity are impossible to overcome but must be acknowledged (Cubbon et al., 2010; Murphy et al., 2007; Roberts et al., 2023). The study is limited by the cross‐sectional design. To now build upon the cross‐sectional observations reported here, future studies should investigate the efficacy of interventions to potentially reduce the absolute number of monocytes which migrate and adhere in a SA population.

## 6 CONCLUSION

In conclusion, the current data provide new insight into the mechanisms underpinning elevated CVD risk in SAs. SAs had higher concentrations, transmigration, and adhesion of monocytes compared with WEs, and this was associated with adverse cardiovascular‐inflammatory profiles. Importantly, LE‐SAs had similar monocyte concentrations, transmigration, and adhesion compared with CO‐WEs, corresponding with similar cardiovascular‐inflammatory profiles. This may partly explain why SAs have a higher risk of CVD at lower body mass index than WEs. Future exploration of these mechanisms may give rise to new ethnic‐specific therapeutic targets. This, in synergy with future examination of the role of controlled lifestyle (exercise and diet) interventions on these mechanisms, will help to develop effective therapies to improve cardiometabolic and inflammatory profiles in SAs and reduce CVD risk in this vulnerable group.

## AUTHOR CONTRIBUTIONS

Matthew J. Roberts and Nicolette C. Bishop were involved in the conception and design of the study. Matthew J. Roberts and Alex J. Wadley developed the laboratory methods. Matthew J. Roberts and Malik Hamrouni undertook recruitment and participant testing. Matthew J. Roberts carried out statistical analysis and data presentation. Drafting of the article for important intellectual content was undertaken by Matthew J. Roberts, Malik Hamrouni, Alex J. Wadley, and Nicolette C. Bishop. All authors undertook revision and final approval of the manuscript.

## FUNDING INFORMATION

This research was supported by the National Institute for Health Research (NIHR) Leicester Biomedical Research Centre. The views expressed are those of the authors and not necessarily those of the NHS, the NIHR, or the Department of Health.

## CONFLICT OF INTEREST STATEMENT

None of the authors have any conflicts of interest to disclose.

## CLINICAL TRIALS

ClinicalTrials.gov (NCT04761081).

## Data Availability

Data will be made available through ClinicalTrials.Gov (NCT04761081) once the study is published.
